# Supplementary material for: White Light-Emitting Flexible Displays with Quantum-Dot Film and Greenish-Blue Organic Light-Emitting Diodes
Source: Micromachines (Basel). 2024 Dec 20;15(12):1518. doi: 10.3390/mi15121518 (PMC11727690; doi:10.3390/mi15121518)

White Emitting flexible Displays with Quantum-dots Film and Greenish Blue Organic Light Emitting Diodes

Young Woo Kim[1], Seojin Kim[1], Chaeyeong Lee[1], Joo Hyun Jeong[1], Yun Hyeok Jeong[1], Yuhwa Bak[1], Seo Hyeon Kim[1], Sung Jin Park[1], Ko Eun Ham[1], Doeun Lee[1], Junpyo Song[1], Youngjin Song[1], Seung-Chan Jung[3], Jae-Hee Han[3], Oh Kwan Kwon[2], Sang Jik Kwon[1], Eou-Sik Cho[1*] and Yongmin Jeon[1,4*]

[1]Department of Semiconductor Engineering, Gachon University, 1342 Seongnam-Daero, Soojung-gu, Seongnam City, Gyeonggi-do, 13120, South Korea

[2]InnoQD, Co., LTD., Dunpo−myeon, Asan−si, Chungcheongnam−do, Korea

[3]Department of Materials Science and Engineering, Gachon University, Seongnam 13120, Republic of Korea

[4]Department of Biomedical Engineering, Gachon University, 1342 Seongnam-Daero, Soojung-gu, Seongnam City, Gyeonggi-do, 13120, South Korea

[*] **Corresponding author:** es.cho@gachon.ac.kr (Eou-Sik Cho), yongmin@gachon.ac.kr (Yongmin Jeon)

Supplementary Table

Table S1. Comparison between several different white emission devices

Devices	Maximum luminance	Color temperature	Flexibility	Method for white emission	Reference
QD-OLED	14,638 cd/m^2 @ 7 V	6041 K	○	OLED attach to QD-film	This work
OLED	N/A	3000 K	×	AC driving	[10]
OLED	N/A	4860 K	×	Tandem	[12]
OLED	15,000 cd/m^2 @ N/A	3000 K	×	Tandem	[13]
OLED	10,200 cd/m^2 @ 40 V	4660 K	×	Tandem	[14]
OLED	30,000 cd/m^2 @ 8.5 V	N/A	○	Parallel-Stack (Tandem)	[15]
OLED	5,219 cd/m^2 @ 15 V	4500 K	×	Organic material engineering	[19]
OLED	400 cd/m^2 @ > 15 V	5200 K	×	Organic material engineering	[20]
OLED	N/A	6500 K	×	Pixel miniaturization	[22]
OLED	4,000 cd/m^2 @ 20 V	5100 K	×	Organic material engineering	[23]
QD-OLED	964 cd/m^2 @ 8 V	2574 K	×	Spin coated QD solution thin film	[24]
QD-OLED	4133 cd/m^2 @ 12 V	6200 K	×	Spin coated QD solution thin film	[25]
Perovskite QD-OLED	49,000 cd/m^2 @ 12 V	5600 K	×	Spin coated QD solution thin film	[26]

Supplementary Figures

Figure S1. Characteristics of blue and greenish blue OLEDs: (a) luminance vs voltage characteristics, **(b)** current density vs voltage characteristics, **(c)** spectral characteristics.

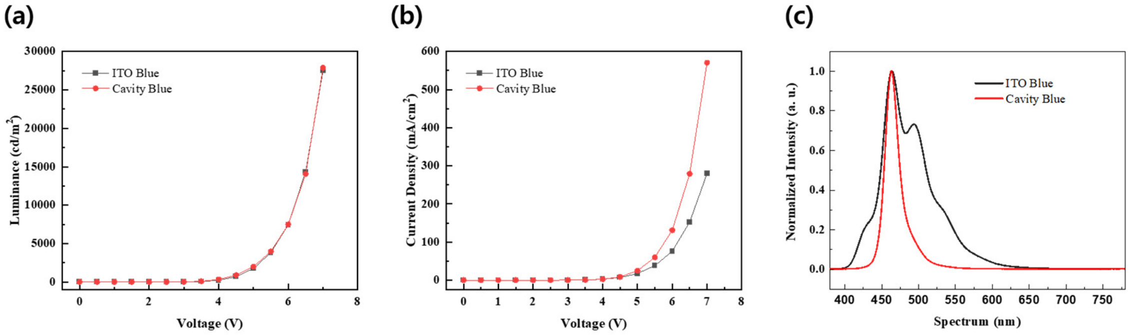

Figure S2. CIE 1931 coordination against ITO based and cavity based QD-OELDs.

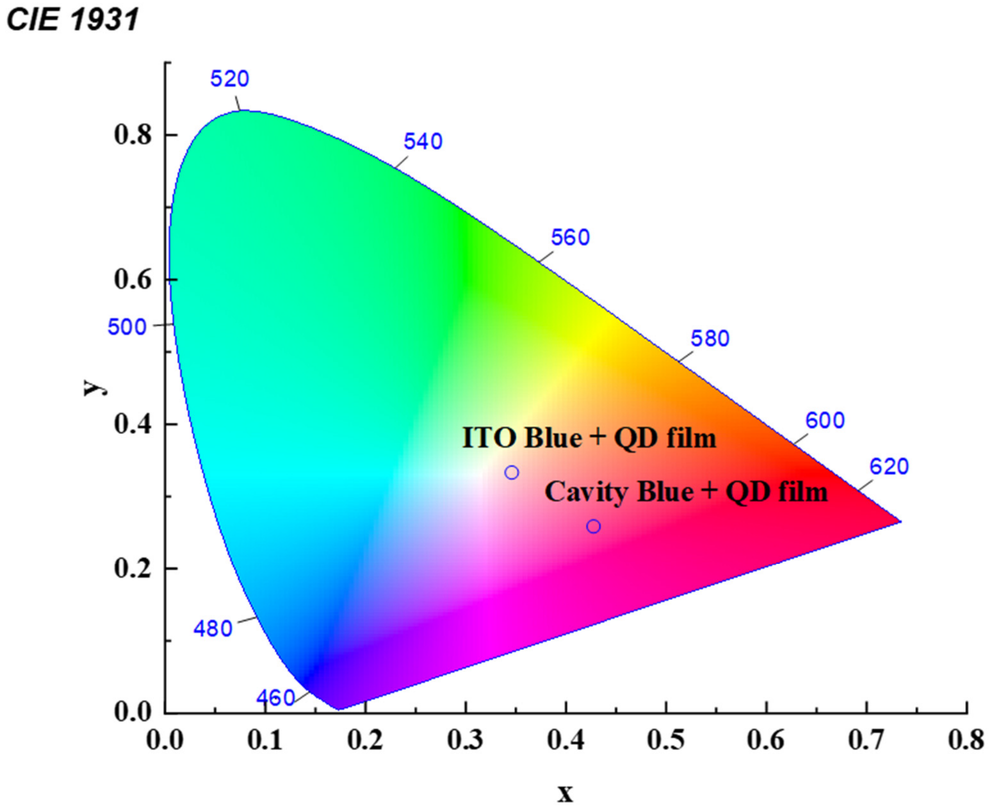

Figure S3. Current density vs voltage characteristics of various QD-OLEDs.

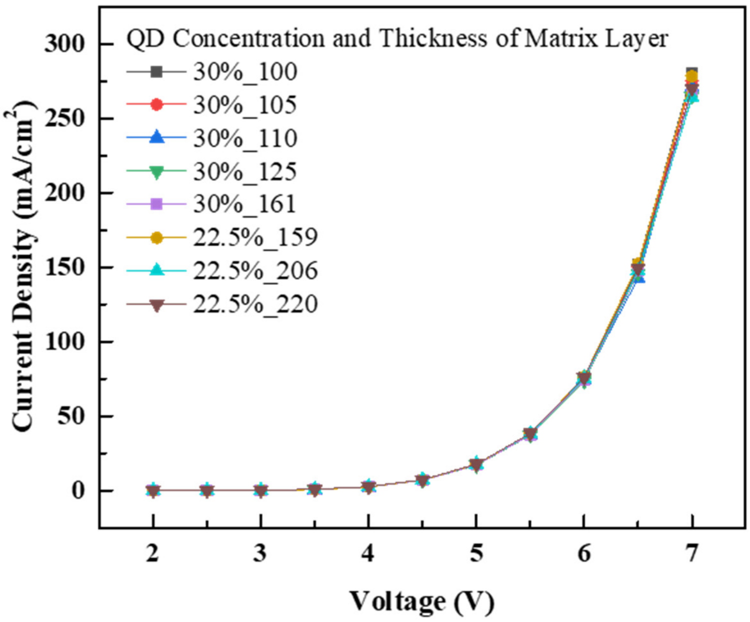

Supplement: Supplementary file 1 [file micromachines-15-01518-s001.zip › micromachines-3370901-supplementary.pdf]
